# Understanding suicidality in adolescents and young adults at clinical high risk for psychosis: a narrative review on risk factors and clinical insights

**DOI:** 10.3389/fpsyt.2025.1580646

**Published:** 2025-06-19

**Authors:** Maria Pontillo, Cristina Di Vincenzo, Michelangelo Di Luzio, Francesco Demaria, Barbara D’Aiello, Ilaria Bertoncini, Massimo Apicella, Milena Labonia, Gino Maglio, Roberto Averna, Stefano Vicari

**Affiliations:** ^1^ Child and Adolescence Neuropsychiatry Unit, Bambino Gesù Children’s Hospital, Istituto di Ricovero e Cura a Carattere Scientifico (IRCCS), Rome, Italy; ^2^ Department of Psychology, Catholic University of the Sacred Heart, Milan, Italy; ^3^ Department of Life Sciences and Public Health, Catholic University of the Sacred Heart, Rome, Italy

**Keywords:** self-injurious behaviors, suicidality, adolescents, clinical-high risk, psychosis

## Abstract

The prodromal stage of psychosis, referred to as the Clinical High Risk (CHR) phase, represents a critical period of heightened vulnerability to suicidality. Although suicidality is highly prevalent in CHR for psychosis (CHR-P) populations, research on this topic remains limited, often focusing more on the prevalence rates rather than the clinical implications. In this review, covering the past decade, we examined the prevalence and clinical significance of suicidality in adolescents and young adults at CHR-P. Our findings suggest that suicidality in CHR individuals arises from a complex interplay of depressive symptoms and both negative and positive symptomatology. Additionally, psychosocial stressors such as perceived stigma and discrimination further exacerbate suicide risk. Key risk factors include prior suicide attempts, impaired social functioning, psychiatric comorbidities, and stigma-related distress. Furthermore, anhedonia and suspiciousness emerged as independent predictors of suicidality. Suicidality rates vary by context, with higher prevalence in community-recruited CHR samples than in help-seeking individuals. This review highlights the need for a multidimensional suicide prevention approach, integrating early identification, comprehensive assessment, and targeted interventions. Future research should refine diagnostic tools, clarify the clinical trajectory from CHR to psychosis, and develop tailored intervention strategies to mitigate suicide risk in this population.

## 1 Introduction

Psychotic disorders, including schizophrenia, are strongly associated with heightened suicidality, particularly during the early stages of the illness (1–3). Compared to the general population, young adults with schizophrenia are at a 13-fold higher risk of suicide (4), with approximately 4.9% ultimately dying by suicide (1). Non-suicidal self-harm is also prevalent in this population, with a lifetime prevalence of 29.9% (5). In this review, we refer to suicidality that encompasses a spectrum of thoughts, behaviors, and tendencies related to suicide. Indeed, according to the UK’s National Institute for Health and Care Excellence (NICE) guidelines (6), suicidality is part of a broader category of self-harm behaviors, defined as “any act of self-poisoning or self-injury, irrespective of underlying intent.” This includes completed suicides, suicide attempts, suicidal planning, and non-suicidal self-injury. Suicidal ideation specifically refers to thoughts of ending one’s life, while suicidal planning involves the formulation of a method or plan to commit suicide. Suicide attempts are characterized by self-harming behaviors executed with at least partial intent to die, whereas non-suicidal self-injury refers to self-harming acts without intent to die.

Suicidality is especially common during the early prodromal stages of psychosis, particularly as the illness transitions to full-blown psychosis (7).

The prodromal phase, also referred to as “Clinical High Risk” (CHR), “Ultra High Risk” (UHR), or “At Risk Mental State” (ARMS), leads to a full psychotic disorder in approximately 20% of cases (8). To better categorize these risk states, two main approaches are currently used: the “Clinical High Risk” (CHR) model (9) which focuses primarily on attenuated psychotic symptoms (APS), and the “basic symptoms” approach (10). The CHR criteria include: (1) Attenuated Psychotic Symptoms (APS), characterized by subthreshold positive symptoms; (2) Brief Limited Intermittent Psychotic Symptoms (BLIPS), involving transient psychotic episodes that spontaneously remit within a week; and (3) Genetic Risk and Functioning Deterioration Syndrome (GRFD), involving a family history of psychosis in first-degree relatives or schizotypal personality disorder, coupled with functional decline lasting for one month or less (11).These criteria were specifically designed to identify individuals at imminent risk of developing psychosis, particularly those likely to experience a first episode within 12 months (12). On the other hand, the criteria based on basic symptoms, such as the cognitive-perceptive basic symptoms (COPER) and cognitive disturbances (COGDIS) (10), aim to detect the potential for psychosis at the earliest stages of its development, ideally before any significant functional impairments occur (10).

Also, negative symptoms, characterized by the reduction or absence of normal emotional and behavioral functions, are highly prevalent in individuals at CHR-P. They are generally categorized into two domains: motivation-related deficits, including avolition, anhedonia, withdrawal and expression-related deficits, such as blunted affect and alogia. These symptoms are critical prognostic indicators, significantly affecting real-life functioning, quality of life, and treatment outcomes (13–15). Studies show that negative symptoms, particularly avolition and social amotivation, strongly predict functional impairments and the risk of transition to psychosis (16, 17). Even when attenuated positive symptoms improve, a large proportion of CHR-P individuals experience persistent functional deficits linked to negative symptoms (18), highlighting the need for early identification and intervention.

Within one year, approximately 15.9% to 19.3% of individuals meeting CHR-P criteria transition to psychosis (19). It was observed that the high levels of suicidality in CHR-P populations might precede the onset of frank psychosis (20, 21). Indeed, during this transitional period, estimates indicate that 66% of young adults CHR (aged 8–40 years) engage in self-injurious behaviors, while 26% report suicidal ideation, 18% attempt suicide and lifetime self-harm behavior is 49% figures comparable to those observed in first-episode psychosis (FEP) samples (2). Concord, a high prevalence of suicidal ideation among CHR-P adolescents (13–18 years) was found with 67.5% of CHR-P adolescents had suicidal ideation, and 18.5% to severe degree (3). This increase in suicidality may be associated with several disease-related factors, including the self-awareness of prodromal decline, the rapid onset of frank psychotic symptoms, and heightened anxiety and mood instability (7). In addition, psychotic symptoms in these age groups are associated with increased long-term rates of self-harming behaviors (2, 22).

Despite the critical importance of understanding suicidality during the early stages of psychosis, available data on this topic remain limited. Previous studies have revealed several limitations, including the use of unreliable tools for identifying and assessing self-harm behaviors and a lack of validated instruments for determining Clinical High Risk for Psychosis status. For example, in these studies, CHR-P status was often evaluated using self-report questionnaires or non-specific diagnostic tools for global psychopathology, rather than structured interviews that are considered gold-standard instruments for CHR-P assessment according to EPA guidelines (23). Moreover, despite the complexity and specificity of the clinical picture and the functional impairment of CHR-P adolescents, a few studies have been conducted on this subgroup. Overall, most studies and meta-analyses on this topic have primarily focused on prevalence data. To overcome these limitations, we conducted a narrative review covering the past 10 years, focusing on studies that utilized standardized tools for defining CHR-P status. With this purpose, our narrative review aims not only to examine the prevalence of suicidality within the CHR-P population, but also to identify potential risk factors contributing to the vulnerability to suicidality in this clinical population. This is particularly concerning in order to implement therapeutic programs focused on prevention and early intervention of suicidality in CHR-P population.

## 2 Methods

The current study consists of a narrative review of the literature published between January 2014 and February 2025.

### 2.1 Search strategy

An electronic database search via PubMed was managed to find all included studies. Two different types of algorithms were used for the research: (Self-Injurious Behaviors) OR (suicidal ideation) OR (suicidal attempts) OR (suicidal planning) AND (ultra-high risk for Psychosis) OR (Clinical High Risk for Psychosis) OR (Attenuated Positive Symptoms). 9 articles were included. The last update of the search was in February 2025.

### 2.2 Inclusion and exclusion criteria

The included studies examine the clinical manifestations and risk factors associated with Self-Injurious Behaviors in patients classified as Clinical High Risk for Psychosis (CHR-P) within samples of young adults aged 7 to 35 years. The inclusion criteria comprised original research articles, observational studies, and experimental studies. We included only studies that applied recognized diagnostic criteria for Suicidal Behavior (individuals who have engaged in potentially self-harming acts with at least some degree of intent to die as a result of the act) and Non-Suicidal Self-Injury-NSSI (intentional, self-inflicted harm to the body, such as causing bleeding, bruising, or pain through actions like cutting, burning, stabbing, hitting, or excessive rubbing, performed in the absence of suicidal intent) as mentioned in the *Diagnostic and Statistical Manual of Mental Disorders, 5th Edition, Text Revision* – DSM-5-TR and for Attenuated Psychotic Syndrome (8, 12, 24–26). Additionally, we included studies that used gold-standard instruments to diagnose Clinical High Risk (CHR), such as the *Structured Interview for Psychosis-Risk Syndromes* (SIPS) (27), the *Comprehensive Assessment of At-Risk Mental States* (CAARMS) (11) and their derivations. The exclusion criteria included studies that focused exclusively on patient samples with current or past affective and non-affective psychoses, those falling outside the scope of interest (e.g., genetic studies, instrument validation, or neuroimaging research), as well as specific article formats (reviews, meta-analyses, commentaries, and letters). Furthermore, studies involving samples with psychotic symptoms induced by substance use or abuse were excluded. A language restriction was applied, limiting the selection to articles published in English.

### 2.3 Selection procedure, data extraction, and data management

**Figure 1:**
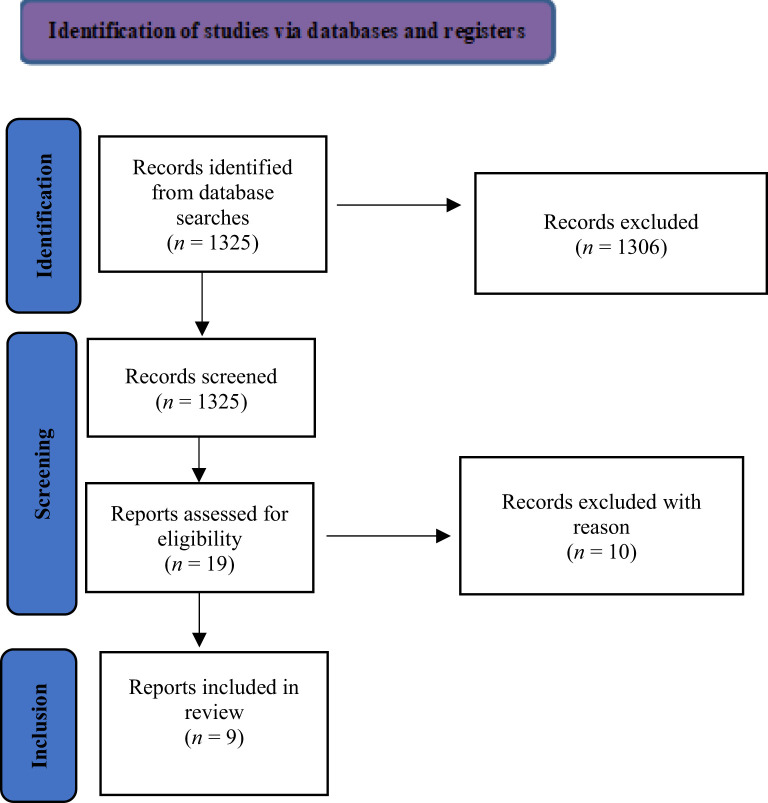
Presents a detailed flow diagram of the study selection process.

The reference lists of crucial articles of interest were thoroughly examined. Eligibility screening of studies was conducted independently by two authors (MP and SV). Potentially eligible studies were identified based on their titles and abstracts. Full texts of the selected articles were subsequently assessed for eligibility. Data regarding study design, sample size, inclusion and exclusion criteria, methodologies, and results were extracted by MP, CDV, IB, DB, ML and BDA. Any discrepancies were resolved through a consensus meeting with additional reviewers (GM, MA, MDL, FD, RA, and ML). The search algorithm yielded a total of 1.325 articles, of which 19 were deemed potentially eligible. Of these, 9 were included for the final analysis, while the remaining 10 were excluded for the reason listed in **Table 1**. Details on the methodology are shown in **Figure 1**.

**Table 1:** Excluded studies and the reasons for their exclusion.

Reason for Exclusion	Study Name
Article format (e.g. review, meta-analyses, commentaries, and letters)	(2, 48–51)
Sample characteristics: only specific population included	(22, 41, 52–55)

## 3 Results

We identified a total of nine studies on suicidality in CHR-P individuals within the selected time frame. These studies explored prevalence, clinical and psychosocial predictors of suicidality in CHR-P individuals. Given the number and heterogeneity of the included studies, a narrative synthesis was conducted to describe, organize, explore, and interpret their findings while assessing their methodological adequacy. Details on the results of the studies and **Table 2**.

**Table 2:** Results of the investigated studies.

Study	Sample	Method(s)	Measures	Results
Haining et al. (28),	130 CHR-P (Mean age: 21.64) 15 FEP (Mean Age: 23.73) 47 CHR-N (Mean age: 22.94) 53 HC (Mean age: 22.42)	Experimental Study	CAARMS SPI-A SIPS/SOPS MINI GF: Role GF: Social	Lifetime suicide attempts were significantly high in CHR-P (29.2%) and FEP (60.0%) compared to CHR-N (8.5%) and HC (0%). As a predictors of suicidal ideation emerged: lifetime suicide attempts (p= .040), lower severity of symptoms as measured by CAARMS (p = .043), compromised social functioning (p=0.22), and higher psychiatric comorbidity (p=0.014).
Pelizza et al. (3),	40 CHR-P 32 FEP 40 Non CHR-P/FEP (13–18 years)	Experimental Study	CAARMS BDI-II	67.5% of CHR-P adolescents experienced suicidal ideation; more prevalence of suicide attempts in the CHR-P group compared to the non- CHR-P/FEP groups (17.5% vs. 2.5%).
Pelizza et al. (29),	180 CHR-P (12–25 years)	Experimental Study	CAARMS BPRS PANSS GAF	At baseline, 51.1% of participants were classified as CHR-P/SI+ due to the presence of current suicidal ideation; 48.9% were included in the CHR- P/SI- sub-group without current suicidal ideation. CHR-P/SI+ individuals had a significantly higher prevalence of past suicide attempts (*p* = 0.002). Over the 2-year follow-up, an increase in incidence of suicide attempts was found, from 3.3% at T1 to 7.2% at T2. A significant longitudinal reduction in suicidal ideation severity was documented in CHR-P sample (*p* = 0.0001) and the improvement in disorganization symptoms was the strongest predictor of decreased suicidal ideation over time (p=0.005).”
Xu et al. (30),	172 individuals (13–35 years) with CHR-P or risk of bipolar disorder.	Experimental Study	HRSD PANSS PDD	Stigma stress is a significant predictor of suicidal ideation among young individuals at risk of psychosis (p =0.02)
Poletti et al. (31),	96 CHR-P 146 FEP 96 CAARMS- (13–35 years)	Experimental Study	CAARMS BDI-II	The results indicated a correlation between anhedonia and suicidal ideation across CHR-P, FEP, and CAARMS- (p=0.000)
Bang et al. (32),	53 HCs 74 CHR-P (15–35 years)	Experimental Study	SIPS MADRS SIQ	CHR-P exhibited higher levels of suicidal ideation and more severe depressive symptoms compared to HCs (p <.001). Suspiciousness or persecutory ideas increase suicidal ideation among CHR-P, regardless of the severity of their depressive symptoms (p= .0.16).
Monducci et al. (34),	17 FEP 22 CHR-P 45 CHSC (13–18 years)	Experimental Study	K-SADS-PL SIPS/SOPS EASE GAF GF: Role GF: Social	Suicidal risk was present in 81.8% of CHR-P, 70.6% of FEP and 28.9% of CHSC. It was significantly higher in FEP/CHR-P than in CHSC (p < 0.001), but did not differ between FEP and CHR-P. Suicidal risk correlated positively with positive symptoms (p = 0.013), negative symptoms (p = 0.032), general symptoms (p = 0.009), and global functioning (p < 0.001). The EASE total score also strongly correlated with SR (p < 0.001).
D’Angelo et al. (35),	40 CHR-P 25 PD 21 HC	Experimental Study	KSADS-PL SIPS/SOPS SBQ-R	Higher mean on SBQ-R scores for CHR and PD compared to the HC (p = 0.008 and p = 0.001 respectively). Within the CHR, Odd Behavior and Appearance and Dysphoric Mood showed a significant correlation with the severity of suicidal behavior (p = 0.005; p = 0.001).
Thompson et al. (43),	569 subjects (12–18 years) hospitalized in a psychiatric ward for self-directed or other-directed aggressiveness	Experimental Study	ChIPS PRIME SIQ-Jr	Suicidal ideation and lifetime suicidal attempts showed positive correlation with depression (p<.01), full-blown psychosis (p<.05), anxiety (p<.01) and post-traumatic stress disorder (p<.01).

CAARMS, Comprehensive Assessment of At-Risk Mental States; SPI-A, Schizophrenia Proneness Instrument, Adult version; MINI, Mini-International Neuropsychiatric Interview; GF, Global Functioning Social and Role Scale; BDI-II, Beck Depression Inventory-II; BPRS, Brief Psychiatric Rating Scale; PPD, Perceived Devaluation-Discrimination Questionnaire; HRSD, Hamilton Rating Scale for Depression; PANSS, Positive and Negative Syndrome Scale; SIPS, the Structured Interview for Psychosis-risk Symptoms; MADRS, Montgomery-Åsberg Depression Rating Scale; SIQ, Reynolds Suicidal Ideation Questionnaire; KSADS-PL, Schedule for Affective Disorders and Schizophrenia for School-Age Children—Present and Lifetime Version; EASE, Examination of Anomalous Self-Experience; GAF, Global Assessment of Functioning; SBQ-R, Suicide Behaviors Questionnaire-Revised; ChIPS, Childhood Inventory of Psychiatric Syndromes; PRIME, PRIME Screen-Revised; SIQ-Jr, Suicidal Ideation Questionnaire- Junior.

### 3.1 Prevalence of suicidality in CHR-P individuals: variability across contexts

In the last ten years, few studies have been conducted on the prevalence of Suicidality in CHR-P, revealing progressively increasing rates.

In our narrative review, we have included only two study with focus on prevalence of Suicidality in CHR-P.

Haining (28) aimed to assess the prevalence of suicidal tendencies and self-harm among 130 participants classified as Clinical High Risk for Psychosis (CHR-P), along with 15 individuals experiencing first-episode psychosis (FEP), 47 participants not meeting CHR-P criteria (CHR-N), and 53 healthy controls (HCs). The study utilized online screening questionnaires, including the 16-item Prodromal Questionnaire (PQ-16) and a nine-item Anomalies of Perception and Cognition (PCA) scale to evaluate basic symptoms. Participants who did not meet CHR-P criteria but had psychiatric comorbidities (CHR-N) were included alongside the healthy control group. CHR-P criteria were established using the Comprehensive Assessment of At-Risk Mental States (CAARMS), a semi-structured clinical interview comprising 27 items, each rated on intensity and frequency/duration scales ranging from 0 to 6. CHR-P criteria were also evaluated using the Cognitive Disturbances (COGDIS) and Cognitive Perceptive Basic Symptoms (COPER) items from the Schizophrenia Proneness Instrument, Adult version (SPI-A), a semi-structured interview with six subscales (kappa = 0.81). Current and lifetime suicidal tendencies and self-harm were assessed using the six-item suicidality module of the Mini-International Neuropsychiatric Interview (MINI), a structured diagnostic interview, alongside questions from the suicidality and self-harm subscale of the CAARMS. The prevalence of suicidal tendencies and self-harm in CHR-P and FEP samples was significantly higher than in CHR-N and HC groups. Suicide attempts were reported by 29.2% of CHR-P and 60.0% of FEP participants, compared to 8.5% of CHR-N and 0% of HC participants. Current suicidal ideation was noted in 34.6% of CHR-P, 19.1% of CHR-N, and 73.3% of FEP participants. Current intentions to self-harm were reported by 28.5% of CHR-P, 8.5% of CHR-N, and 60% of FEP participants.

Pelizza (3) examined the prevalence of suicidality among 40 adolescents classified as Clinical High Risk for Psychosis (CHR-P) (aged 13–18 years) recruited through the “Reggio Emilia At-Risk Mental States” (ReARMS) project. The study included a comparison group of 32 adolescents with first-episode psychosis (FEP) and 40 non-CHR-P adolescents. Suicidal ideation was assessed over a two-year follow-up period, considering the incidence of suicide attempts and completed suicides. The CHR-P and psychosis were evaluated using the Comprehensive Assessment of At-Risk Mental States (CAARMS). Depression severity was measured with the Beck Depression Inventory-II (BDI-II), a 21-item self-report questionnaire utilizing a 4-point Likert scale. Suicidality was assessed using specific items from the CAARMS (item 7.3: “Suicidality/Self-Harm”) and the BDI-II (item 9: “Suicidal thoughts or wishes”). The results indicated that 67.5% of CHR-P adolescents reported experiencing suicidality, with 18.5% exhibiting severe levels. Additionally, suicidality were more prevalent in the CHR-P group compared to the non-CHR-P and FEP groups (17.5% vs. 2.5%). The severity of suicidality in the CHR-P group remained stable at the one-year follow-up but showed a decrease by the two-year mark. Importantly, no suicides occurred during the follow-up period.

Pelizza et al. (29) also conducted a recent longitudinal study to assess the baseline prevalence and two-year incidence of suicidal thoughts and behaviors in a cohort of 180 individuals at clinical high risk for psychosis (CHR-P). The study also examined the temporal stability of suicidal ideation (SI) and its associations with treatment outcomes, sociodemographic characteristics, and clinical variables. CHR-P and psychosis were defined using the Comprehensive Assessment of At-Risk Mental States (CAARMS), while current SI was identified by a score of ≥3 on item 4 of the Brief Psychiatric Rating Scale (BPRS). Psychopathology was assessed using the Positive and Negative Syndrome Scale (PANSS), and global functioning was measured through the Global Assessment of Functioning (GAF) scale. At baseline, 92 participants (51.1%) were classified as CHR-P/SI+ due to the presence of current suicidal ideation, and the remaining 88 (48.9%) were included in the CHR- P/SI- sub-group (no current suicidal ideation). Compared to the CHR-P/SI− subgroup, CHR-P/SI+ individuals had a significantly higher prevalence of past suicide attempts (p = 0.002). Over the 2-year follow-up, a progressive increase in the cumulative incidence of suicide attempts was observed, from 3.3% at T1 to 7.2% at T2. Importantly, a significant longitudinal reduction in suicidal ideation severity was documented across the total CHR-P sample (p = 0.0001). Linear regression showed that improvement in disorganization symptoms was the strongest predictor of decreased suicidal ideation over time (p=0.005).

### 3.2 Clinical and psychosocial predictors of suicidality in CHR-P

Identifying predictors of suicidality in individuals at Clinical High Risk for Psychosis (CHR-P) is essential for effective risk management and the development of prevention strategies.

Haining (28) aimed not only to assess the prevalence of suicidality but also to identify its predictors within the Clinical High Risk for Psychosis (CHR-P) group. The study found that a higher number of lifetime suicide attempts (p = .040), lower symptom severity as measured by the Comprehensive Assessment of At-Risk Mental States (CAARMS) (p = .043), compromised social functioning (p = .22), and higher psychiatric comorbidity (p = .014) emerged as significant predictors of suicidality.

Xu et al. (30), investigated whether the perception of public stigma and the cognitive appraisal of stigma as a stressor (termed stigma stress) could predict suicidality among individuals at risk of psychosis over the course of one year. The participant sample consisted of 172 individuals aged between 13 and 35 who were identified as being at high or clinical high risk for psychosis or bipolar disorder. At the one-year follow-up, data were available for 73 participants. Perceived stigma was measured using the 12-item Perceived Devaluation-Discrimination Questionnaire, with scores indicating higher perceived stigma (baseline: M = 3.6, SD = 1.0; follow-up: M = 3.5, SD = 1.0; Cronbach’s alpha: baseline = 0.92; follow-up = 0.88). Stigma stress was assessed using the 8-item Stigma Stress Scale, which comprises two 4-item subscales. Depressive symptoms were evaluated using the Hamilton Rating Scale for Depression (HRSD), a clinician-administered assessment tool based on 17 items, excluding the suicidality item (baseline: M = 13.8, SD = 6.9; follow-up: M = 9.4, SD = 5.9). Suicidality was assessed using a single item from the HRSD, scored from 0 to 4, allowing categorization based on the presence or absence of suicidal thoughts. Positive and negative symptoms of psychosis were measured using the Positive and Negative Syndrome Scale (PANSS) (positive symptoms: baseline: M = 12.5, SD = 4.2; follow-up: M = 10.9, SD = 4.3; negative symptoms: baseline: M = 13.5, SD = 5.4; follow-up: M = 12.2, SD = 6.4). Insight was also evaluated through the PANSS insight item, which assesses lack of insight and judgment (baseline: M = 1.5, SD = 0.9; follow-up: M = 1.4, SD = 0.8). Statistical analyses, including logistic regression, were conducted to examine the associations between these variables and suicidality over the one-year follow-up period. Results indicated that, out of the 73 participants, 17 consistently reported experiencing suicidal thoughts at both the beginning and end of the study, while 46 individuals maintained a consistent lack of suicidality throughout the study period. Additionally, 22 participants reported suicidal thoughts at the outset but not at the conclusion, and 5 participants reported suicidal thoughts after one year. An increase in stigma stress was observed between the baseline and follow-up assessments (Mann-Whitney U = 203, p = 0.003). Importantly, the increase in stigma stress was significantly associated with suicidality at follow-up (p = 0.02), whereas an increase in the perception of public stigma was not significantly correlated with suicidality.

Poletti et al. (31) aimed to investigate the association between anhedonia and suicidality over a two-year follow-up period among individuals with First Episode Psychosis (FEP) and those at Clinical High Risk for Psychosis (CHR-P). The study analyzed 338 participants aged between 13 and 35 and enrolled in the “Reggio Emilia At-Risk Mental States” (ReARMS) protocol, divided into three groups: 96 CHR-P individuals, 146 with first-episode psychosis (FEP), and 96 without clinical risk (CAARMS). The psychopathological assessment was conducted using the Comprehensive Assessment of At-Risk Mental States (CAARMS) and the Beck Depression Inventory-II (BDI-II). The CAARMS, a structured clinical interview, evaluates various psychopathological dimensions, including depression severity, utilizing a 7-point Likert scale. It examines depressive mood, hopelessness, motivation, appetite, sleep continuity, and future perspective over a 12-month period. Anhedonia levels were assessed using the Anhedonia subscale of the BDI-II, which encompasses items related to loss of pleasure, interest, energy, and sexual interest. The risk of suicidality was evaluated using item 9 of the BDI-II, with scores of ≥1 on this item showing a significant association with the total score of the Beck Scale for Suicidal Ideation. The results demonstrated a significant correlation (p = 0.000) between anhedonia and suicidality across all three subgroups (CHR-P, FEP, and CAARMS-), even after controlling for depressive symptoms. These findings indicate that anhedonia may serve as a significant risk factor for suicidality in individuals at clinical risk or those with psychiatric disorders.

Bang et al. (32) conducted a study to examine the influence of attenuated positive symptoms on suicidality in individuals classified as being at Clinical High Risk for Psychosis (CHR-P). The primary hypothesis posited that CHR-P individuals would manifest a higher intensity of suicidality in comparison to healthy controls (HCs), and that experiences of persecutory delusions and hallucinations would serve as significant risk factors for suicidality, even after controlling for comorbid depressive symptoms within the CHR-P cohort. The participant sample comprised 74 CHR-P individuals and 53 HCs, aged between 15 and 35 years. Healthy controls were recruited via an online community advertisement, whereas CHR-P participants were referred through the Clinic FOR YOU associated with the Green Program for Recognition and Prevention of Early Psychosis (GRAPE) at Severance Hospital, part of the Yonsei University Health System in Seoul, Republic of Korea. At baseline, the severity of attenuated positive symptoms in CHR-P participants was assessed using the five domains of the Structured Interview for Psychosis-risk Symptoms (SIPS), which include: perceptual disturbance, unusual thought content, magical or delusional beliefs, disorganized thinking, and excessive suspicion. Symptoms of depression were evaluated using the Montgomery-Åsberg Depression Rating Scale (MADRS), a structured interview instrument composed of 10 items, which demonstrated excellent internal consistency (Cronbach’s alpha = 0.95). The intensity of suicidality was quantified utilizing the Reynolds Suicidal Ideation Questionnaire (SIQ), a 7-point Likert scale ranging from 0 (“I never had this thought”) to 6 (“Almost every day”), which also exhibited high internal consistency (Cronbach’s alpha = 0.98). The results indicated that CHR-P individuals reported significantly elevated levels of suicidality and greater severity of depressive symptoms relative to HCs (p <.001). All CHR-P participants presented at least one attenuated or transient positive symptom, meeting the diagnostic criteria for either Attenuated Positive Symptom Syndrome (APSS) or Brief Intermittent Psychotic Syndrome (BIPS). Specifically, the prevalence of positive symptoms included: 74.3% with unusual thought content, 81.1% with suspiciousness or persecutory ideas, 6.8% exhibiting grandiose ideation, 48.6% experiencing perceptual abnormalities, and 20.3% displaying disorganized communication. Importantly, the presence of suspiciousness or persecutory ideas was significantly associated with increased suicidality among CHR-P participants, independent of the severity of depressive symptoms (p = .016).

### 3.3 Suicidality in adolescents at clinical high risk for psychosis: key factors

A lot of research conducted so far on suicidality in individuals with Clinical High Risk for Psychosis has focused on mixed-age clinical samples, including adolescents and young adults. However, adolescence is a critical period for hormonal changes, brain development and the acquisition of metacognitive and social skills.

Therefore, it is important to better understand the clinical significance of suicidality in CHR-P patients during the adolescent developmental period, in which early interventions are necessary, and clinical manifestations can be peculiar to the age group (33).

Monducci et al. (34) investigate suicidality in CHR-P adolescents, comparing them with peers experiencing a FEP and clinical help-seeking controls (CHSC) with other psychiatric disorders. Additionally, the research explored the relationship between suicidality and self-disorders (SDs), which are anomalies in subjective experience. The sample included 95 adolescents aged 13 to 18 years. Participants were recruited from the Child and Adolescent Neurology and Psychiatry Department of the University-Hospital Policlinico Umberto I and “Sapienza” University of Rome, a third-level referral center. They were divided into three groups: 17 FEP, 33 CHR-P, and 45 CHSC. Psychopathological assessment was conducted using several tools. Diagnoses were established through the Kiddie-Schedule for Affective Disorders and Schizophrenia-Present and Lifetime Version (K-SADS-PL) based on DSM-5 criteria. The Structured Interview for Prodromal Syndromes/Prodromal Symptom Scale (SIPS/SOPS) identified CHR-P individuals by assessing dimensions such as positive, negative, disorganized, and general symptoms. Suicide risk was evaluated via clinical interviews using a Likert scale ranging from 0 (no risk) to 4 (very severe risk). The Examination of Anomalous Self-Experience (EASE) assessed SDs across five domains, including cognition, self-awareness, and existential reorientation. Functional levels were measured using the Global Assessment of Functioning (GAF) and Global Functioning Role/Social Scales (GF: SS/RS). The findings revealed that 54.7% of the overall sample was at risk of suicide, with the prevalence being highest among CHR-P adolescents (81.8%), followed by FEP (70.6%) and CHSC (28.9%). Statistical analysis showed significant differences in SR between FEP/CHR-P groups and CHSC (p < 0.001), though no significant difference emerged between FEP and CHR-P. Moreover, SR was positively correlated with positive symptoms (p = 0.013), negative symptoms (p = 0.032), general symptoms (p = 0.009), and global functioning (p < 0.001). The EASE total score also strongly correlated with SR (p < 0.001). In conclusion, CHR-P adolescents exhibited a significantly higher SR compared to non-CHR-P clinical controls, similar to that of FEP adolescents. SDs were strongly associated with SR and could serve as an important factor for early prognostic stratification.

D’Angelo et al. (35) conducted a case-control study with a sample of 86 young participants (mean age 12.2 years), comprising 40 clinical high-risk (CHR) outpatients, 25 outpatients with a psychotic disorder (PD), and 21 healthy controls (HC). The study aimed to compare the intensity and frequency of suicidality among the three groups and to further investigate the relationship between suicidality and psychotic-like symptoms in CHR adolescents. The authors assessed the participants using the Schedule for Affective Disorders and Schizophrenia for School-Age Children—Present and Lifetime Version (KSADS-PL), to identify past or present psychotic disorders; the Structured Interview for Prodromal Symptoms (SIPS), to detect prodromal psychotic symptoms; and the Suicide Behaviors Questionnaire-Revised (SBQ-R), a 4-item self-report questionnaire (Cronbach’s alpha = 0.64), to assess past, present, or future suicidality. The findings revealed higher SBQ-R scores for the CHR and PD groups compared to the HC group (p = 0.008 and p = 0.001, respectively), with no significant differences between CHR and PD patients. Specifically, within the CHR group, the symptoms of Odd Behavior and Appearance (item D1 on the SIPS) and Dysphoric Mood were significantly correlated with the severity of suicidality (p = 0.005; p = 0.001).

In a retrospective study, Thompson et al. explored the relationship between suicidal thoughts and behaviors (STB) and psychosis-risk symptoms in a sample of adolescent inpatients. The study included 569 participants (aged 12–18 years) who were hospitalized in a psychiatric ward due to self-directed or other-directed aggressive behaviors. Several assessment tools were employed, including the Childhood Inventory of Psychiatric Syndromes (ChIPS), an interview designed for diagnosing psychiatric disorders; the PRIME Screen-Revised (PRIME), a self-report measure for screening psychosis-risk symptoms; the Suicidal Ideation Questionnaire-Junior (SIQ-Jr), a 15-item self-report questionnaire for assessing suicidal ideation in adolescents (Cronbach’s alpha = 0.95); and a self-report question regarding lifetime suicide attempts. The findings showed that, among the STB variables, both suicidal ideation and lifetime suicide attempts were positively correlated with symptoms of depression (p <.01), full-blown psychosis (p <.05), anxiety (p <.01), and post-traumatic stress disorder (PTSD) (p <.01). Specifically, several items on the PRIME (e.g., “Odd or unusual things going on,” “Something interrupting or controlling me,” “Superstitious beliefs,” “Confusing real life with imagination/dreams,” “Mind-reading,” “People planning to hurt me,” “Special natural or supernatural abilities,” “Mind playing tricks on me,” “Hearing mumbling or talking,” “Thoughts being said out loud,” and “Feeling like going crazy”) were positively correlated with suicidality (p <.01). However, it is important to note that PRIME items were also associated with depression, anxiety, and PTSD, in addition to identifying psychosis-risk symptoms.

## 4 Discussion

In our narrative review, we explore the predictors and risk factors that may contribute to suicidality in individuals at Clinical High Risk for Psychosis (CHR-P).

In the following sections, we will analyze the results obtained from recent studies, highlighting critical findings that may inform targeted prevention and intervention strategies for suicidality in CHR-P individuals.

### 4.1 Prevalence of suicidality in CHR-P individuals: variability across contexts

Suicidality represents a significant issue in individuals at clinical high risk for psychosis (CHR-P), with prevalence rates varying considerably across different populations and study settings. Specifically, suicidality has been reported in 34.6% of young adults with CHR-P recruited from community settings and in as many as 73.3% of individuals experiencing a first episode of psychosis (FEP), compared to 19.1% of those with other psychiatric comorbidities and 0% in non-clinical control groups (28). These findings underscore the heightened vulnerability to suicidality in CHR-P individuals, particularly in those transitioning to full-blown psychosis.

When considering CHR-P populations specifically, prevalence estimates of suicidality exhibit notable variability depending on the enrollment context. Among help-seeking CHR-P individuals, reported rates range between 8.6% and 18% (2, 36), which is markedly lower than those observed in community-recruited CHR-P samples. This discrepancy suggests that engagement with clinical services may offer protective benefits, potentially through early identification, access to therapeutic interventions, and enhanced coping mechanisms.

However, these differences must be interpreted with caution, as methodological variations across studies, including sample characteristics, assessment tools, and recruitment strategies, may influence prevalence estimates. It is also plausible that CHR-P individuals identified in community settings experience higher levels of untreated distress, lower symptom awareness, and reduced access to psychological support, all of which may contribute to an increased risk of suicidality. Conversely, help-seeking CHR-P individuals may demonstrate greater resilience and social support, factors that could mitigate suicide risk. Further research is warranted to elucidate these distinctions and to determine whether tailored interventions could address the specific needs of community-identified CHR-P individuals who may be at an elevated risk for suicidality.

### 4.2 Clinical and psychosocial predictors of suicidality in CHR-P

Recent research has identified several key clinical and psychopathological predictors of suicidality in individuals at clinical high risk for psychosis (CHR-P), with particular attention to depressive symptoms, basic symptoms, and negative symptomatology (e.g., anhedonia, avolition, and apathy). Among these, depressive symptoms have emerged as a crucial factor in predicting suicidality. In their study, Pelizza et al. (3) found that higher levels of depression represented the most significant psychopathological correlate of suicidality in CHR-P individuals. This finding is particularly relevant for two reasons. First, depressive disorders are among the most frequently observed comorbid conditions in CHR-P populations (37, 38), with Dolz et al. (37) reporting that depression was the most prevalent psychiatric diagnosis in individuals meeting CHR-P criteria. Second, this association underscores the clinical relevance of systematically monitoring depressive symptoms in CHR-P individuals, given their potential role in heightening suicide risk.

Beyond depressive symptoms, our findings also highlight the contribution of basic symptoms—subjective disturbances affecting cognition, speech, perception, attention, stress tolerance, and affect regulation—as potential risk factors for suicidality in CHR-P (3). These symptoms may exacerbate distress and functional impairment, increasing vulnerability to suicidal ideation and behavior. Similarly, negative symptoms, particularly anhedonia and avolition, have been implicated in suicide risk. These symptoms, characterized by a profound reduction in the ability to engage with one’s environment and maintain social and emotional functioning, may contribute to psychological distress and suicidality. While Pelizza et al. (3) primarily focused on depressive symptoms as a predictor of suicidality, other studies, such as Poletti et al. (31), highlight the importance of negative symptomatology. In particular, attenuated negative symptoms—often more prevalent than positive symptoms in the prodromal phase of psychosis (39, 40)—may play a crucial role in shaping vulnerability to suicidal risk.

However, the absence of specific and sensitive assessment tools for accurate differential diagnosis between negative symptomatology complicates the differentiation between depressive and negative symptoms, creating diagnostic challenges. This issue is particularly evident in the case of anhedonia, which is defined as a diminished capacity to experience pleasure and engage in rewarding activities. Poletti et al. (31) found that in individuals with first-episode psychosis (FEP), anhedonia predicts suicidality independently of depression severity. This suggests that, in FEP, anhedonia represents a distinct clinical dimension of negative symptomatology, whereas in CHR-P individuals, its association with suicidality appears more closely tied to depressive symptoms. This discrepancy may reflect the evolving clinical trajectory of CHR-P individuals compared to those with FEP.

Longitudinal research is needed to clarify whether depression and negative symptoms independently predict suicidality in CHR-P individuals or whether depression serves as an early indicator of emerging negative symptoms and, ultimately, suicide risk.

Developing refined assessment tools that enable a more accurate differential diagnosis between depressive symptoms and negative symptoms would allow for a better understanding of these associations and their clinical implications.

In addition to depressive and negative symptoms, attenuated positive symptoms have also been linked to suicidality in CHR-P populations. For example, Bang et al. (41) identified suspiciousness as an independent predictor of suicidality, regardless of depressive symptoms. The distress associated with suspiciousness—characterized by unfamiliarity, confusion, and negative interpretations of unusual experiences—may exacerbate psychological distress and contribute to suicidality. Cognitive mechanisms such as catastrophizing, heightened threat perception, and negative self-evaluation may further mediate the relationship between suspiciousness and suicide risk. These findings suggest that suspiciousness not only serves as a potential marker for the transition to psychosis but also represents an important risk factor for suicidality in CHR-P populations.

Beyond clinical factors, suicidality in CHR-P individuals is also influenced by environmental and psychosocial stressors, particularly perceived stigma and discrimination. Xu et al. (30) demonstrated that experiences of stigma following a CHR-P diagnosis significantly predict suicidality. Public stigma, defined as the societal dissemination of negative stereotypes and discriminatory attitudes toward individuals with mental health conditions, does not always provoke a stress response. However, when individuals internalize these stereotypes and identify themselves as mentally ill, stigma becomes a significant source of distress, leading to increased feelings of shame, social withdrawal, avoidance of help-seeking behaviors, diminished quality of life, and heightened hopelessness.

The link between stigma and suicide risk is further supported by recent studies examining the unintended consequences of diagnostic labels such as “Ultra-High Risk” (UHR) and “Attenuated Psychosis Syndrome” (APS) (42). While these terms are intended to facilitate early detection and intervention, they may inadvertently reinforce negative self-perceptions and contribute to psychological distress. Given these findings, future research should explore strategies to mitigate the adverse effects of stigma in CHR-P populations, including psychoeducation, interventions aimed at enhancing resilience, and broader public health efforts to reduce societal stigma surrounding psychosis risk syndromes.

In summary, suicidality in CHR-P individuals is shaped by a complex interplay of clinical and psychosocial factors. Depressive symptoms, negative symptomatology, and suspiciousness are key psychopathological predictors, while perceived stigma and discrimination further compound suicide risk. These findings highlight the need for a multidimensional approach to suicide prevention in CHR-P populations, incorporating early identification, comprehensive assessment, and targeted interventions designed to reduce distress and promote resilience. Future research should focus on refining diagnostic tools, elucidating the clinical trajectory from CHR-P to FEP, and developing tailored intervention strategies to address the unique vulnerabilities of this population.

### 4.3 Suicidality in adolescents at clinical high risk for psychosis: key factors

Adolescence represents a critical developmental stage, both biologically and psychologically, and for individuals at clinical high risk for psychosis (CHR-P), it is a particularly vulnerable period. The incidence of suicidality in this population has been consistently high in recent studies, underlining the urgent need for focused, tailored interventions. Pellizza et al. (3) reported a strikingly high rate of suicidality in CHR-P adolescents seeking help, with 67.5% of individuals showing suicidal thoughts and 18.5% presenting severe suicidality. Furthermore, after a 24-month follow-up, 10.5% of these individuals still exhibited suicidality, highlighting that even with ongoing clinical support, suicidality continues to be a prominent risk. This suggests that while interventions may reduce the intensity of suicidality, they may not be sufficient to eliminate the risk completely. Therefore, ongoing monitoring and intervention are essential.

Our findings reinforce the importance of implementing more comprehensive, individualized approaches to suicide prevention, which go beyond the standard treatment paradigms often utilized for this population. One such approach is the incorporation of psychoeducational interventions focused on enhancing self-awareness and insight. Specifically, helping CHR-P adolescents identify and understand cognitive biases and distorted thinking patterns is crucial for preventing the escalation of suicidality. Programs aimed at fostering a deeper understanding of the anomalous experiences during the prodromal phase of psychosis can help normalize these experiences, thereby reducing the reactive depressive symptoms commonly seen in this stage of psychosis (39). It is essential that these interventions be integrated into the care plan for adolescents at risk, as they can serve to build resilience, enhance coping strategies, and ultimately mitigate the risk of suicide.

The study by Monducci et al. (34) further supports the view that suicidality remains high not only in CHR-P individuals but also in those experiencing first-episode psychosis (FEP). Their findings show that the prevalence of suicidality in both CHR-P (81.8%) and FEP individuals (70.6%) was far higher than in the control group (28.9%), regardless of the severity of suicidality. This reinforces the complexity of the clinical profile in CHR-P individuals, which requires a multidimensional approach for assessing suicide risk. Given the high rates of suicidality observed in both CHR-P and FEP populations, clinicians need to consider a wide range of clinical factors that may interact to increase the risk of suicidality, such as mood disturbances, psychotic symptoms, cognitive impairment, and social stressors.

An in-depth understanding of how various psychopathological dimensions contribute to suicidality is crucial for refining prevention strategies. D’Angelo et al. (35) emphasized the importance of disorganization symptoms, including odd behaviors, unkempt appearance, and dysphoric mood, as contributing factors to suicidality. Disorganized symptoms can exacerbate the distress experienced by CHR-P adolescents, impairing their ability to function socially and emotionally, thereby heightening the likelihood of suicidal thoughts and behaviors. The association between disorganization symptoms and suicidality in CHR-P individuals is also highlighted by Pelizza et al. (29), whose recent study found that, over a two-year period, the improvement of disorganized symptoms emerged as a key predictor of the reduction in suicidal ideation. These findings suggest that targeting disorganization may be critical for effective suicide prevention strategies in the CHR-P population. Moreover, Thompson (43) pointed to the role of depression in suicidality, while also noting that anxiety and PTSD can significantly affect the overall clinical profile of these individuals. Therefore, it is not only depression but also other mental health issues, such as anxiety or PTSD symptoms, that need to be considered when developing suicide prevention strategies for CHR-P adolescents. In this context, it may be of interest to investigate the potential role of factors such as aberrant salience in mediating not only the emergence of positive symptoms (44), but also the onset of suicidality in CHR-P adolescents and young adults presenting with PTSD symptoms.

Our results suggest that, in addition to depressive symptoms, attention should also be given to other aspects of the clinical presentation, particularly positive symptoms like suspiciousness. Bang’s research, which focused on adolescents aged 15–18 years, highlights the significant role of suspiciousness in predicting suicidality. Suspiciousness, often characterized by paranoia or distrust in others, can increase the distress of adolescents who are already grappling with uncertainty about their mental health, thus contributing to the emergence of suicidal thoughts. Moreover, recent studies, including those by Monducci et al. (34), underscore the importance of assessing not only the attenuated psychotic symptoms, such as positive, negative, and disorganized symptoms but also non-psychotic anomalies of self-experience, such as self-disorders. Self-disorders, which involve disturbances in self-awareness or self-coherence, may serve as significant risk factors for suicidality. These experiences of confusion or alienation within the self can lead to feelings of hopelessness, isolation, and despair, which are known to heighten suicide risk.

Given the complexity of suicidality in CHR-P adolescents, it is vital to develop a comprehensive diagnostic approach that takes into account a broad spectrum of symptoms, including depressive symptoms, self-disorders, as well as negative, positive, and disorganization symptoms. These factors should not be considered in isolation but rather as part of an interconnected clinical profile that informs suicide risk stratification. The combination of these various symptom domains—along with a thorough psychosocial assessment—will enable clinicians to identify those at highest risk and intervene proactively.

## 5 Conclusion

Suicidality in individuals at Clinical High Risk for Psychosis (CHR-P) is a complex phenomenon influenced by a combination of clinical, psychosocial, and environmental factors. Our narrative review highlights the significant variability in suicidality rates among different CHR-P populations, with individuals from community settings exhibiting notably higher rates compared to those seeking clinical care. This suggests that early identification and access to therapeutic interventions may offer protective benefits, reducing suicide risk.

Key clinical predictors of suicidality include depressive symptoms, negative symptomatology (such as anhedonia and avolition), and suspiciousness, with each factor contributing to psychological distress and functional impairment. The role of stigma and discrimination further exacerbates suicide risk, highlighting the importance of addressing these psychosocial stressors in suicide prevention efforts.

Given the multifaceted nature of suicidality in CHR-P populations, a multidimensional approach to prevention is essential. This should include not only early identification and comprehensive assessment but also tailored interventions that address both clinical symptoms and environmental stressors. Interventions focused on psychoeducation, enhancing self-awareness, and building resilience are crucial, particularly for adolescents at CHR-P, who are at heightened risk for suicidality.

Future research should aim to refine diagnostic tools, clarify the clinical trajectory from CHR-P to first-episode psychosis (FEP), and explore the effectiveness of targeted interventions that address the unique vulnerabilities of individuals at risk for suicidality. By integrating clinical and psychosocial dimensions into a comprehensive suicide prevention strategy, we can better support individuals at CHR-P and reduce the risk of suicidality during this critical phase.

## 6 Therapeutic implication

In line with our review’s findings, a therapeutic approach in the early stages of mental disorder development must also include the clinical aim of assessing suicidality. This assessment should consider prior suicide attempts, significant individuals in the patient’s life who have previously attempt suicide, cognitive patterns of pessimistic thinking, and personality traits associated with impulsivity and acting out.

Protective factors include strong social support and good relational functioning. Important aspects to consider when evaluating suicidality include also the method chosen by the individual, their access to it, the likelihood that others might discover the suicidal intention, the probability of immediate intervention to prevent the attempt or provide rapid medical assistance afterward, and the support network available. Additionally, since the risk of acting on suicidal thoughts can change within a single day, suicidality risk assessment must be a continuous process rather than a one-time event. Hospitalization is recommended when there is a presence of a suicidal plan, explicit intent to carry it out, poor behavioral control, mental confusion, and lack of social support.

Moreover, in light of our findings, it would be valuable to consider integrating the assessment of suicidality risk within the framework of the clinical staging model of psychotic disorders proposed by McGorry et al. (45). This model offers a refined diagnostic perspective that conceptualizes psychosis as a progressive continuum, ranging from early vulnerability states to chronic, treatment-resistant illness. By delineating specific stages (from Stage 0: increased risk, to Stage 4: severe and persistent illness), the model supports the implementation of targeted, stage-specific interventions.

Specifically, we propose that the evaluation of suicide risk should be an integral component of the assessment process in CHR-P individuals, as described in Stage 1b of **Table 3**. This approach may enable the early initiation of suicide risk prevention strategies, as outlined in the Key Recommendations for Suicide Risk Prevention adopted by Orygen (46) (see **Table 4**).

**Table 3:** Clinical staging model framework for psychosis adopted by McGorry et al., 2007.

Stage	Definition
0	Increased risk of psychotic or severe mood disorder; no symptoms currently
1a	Mild or non-specific symptoms, including mild neurocognitive deficits of psychosis or severe mood disorder; mild functional change or decline
1b	CHR: moderate but subthreshold symptoms, with moderate neurocognitive changes and functional decline to caseness
2	FEP or severe mood disorder; full threshold disorder with moderate-to-severe symptoms, neurocognitive deficits and functional decline
3a	Incomplete remission from first episode of care; could be linked or fast-tracked to Stage 4
3b	Relapse of psychotic or mood disorder with treatment response stabilizing below the highest level of previous functioning after remission.
3c	Multiple relapses, when worsening in clinical extent and impact of illness is objectively present
4	Severe, persistent, or unremitting illness as judged on symptoms, neurocognition, and disability criteria

**Table 4:** Key recommendations for suicide risk prevention adopted by orygen, The National Centre of Excellence in Youth Mental Health, 2016 (46).

Recommendation	Details
Build a Strong Therapeutic Relationship and Alliance	Establish trust and open communication with the patient, demonstrating empathy and understanding. Create a safe and supportive environment where the patient feels comfortable sharing their thoughts and feelings. Set clear and collaborative goals to promote a sense of partnership and empowerment.
Engage Family Members	Involve family members, with the patient’s consent, in specific monitoring tasks.
Identify a Crisis Contact Person	Work with the patient to identify a contact person, including their phone number, to reach out to during a crisis, including the therapist.
Schedule Regular and Frequent Meetings	Ensure that meetings are frequent, regular, and planned early.
Survey the Patient’s Social Network	Identify other supportive figures among family and friends.
Help the Patient Organize Their Time	Assist the patient in organizing their time between sessions.
Develop a 24-Hour Behavioral Plan	Define a behavioral plan for the next 24 hours with the patient, which can be reviewed by the therapist or an accepted clinical figure.
Use Problem-Solving Techniques	Apply problem-solving techniques to both specific concrete situations and the emergence of particularly activating ideations.

## 7 Strengths and limitations

A notable strength of this review is that it represents the first comprehensive analysis specifically addressing suicidality in individuals at Clinical High Risk for Psychosis (CHR-P). Furthermore, this review uniquely extends beyond the examination of suicidality prevalence, offering a detailed exploration of its clinical significance and potential predictive factors across both young adult and adolescent populations. This approach provides a more nuanced understanding of suicidality within the CHR-P cohort, highlighting the need for targeted, evidence-based interventions that consider the distinct clinical profiles of these individuals.

However, several limitations should be considered when interpreting the findings. Inconsistencies in study designs across the included studies, as well as the inclusion of mixed age groups, limited the possibility of performing a quantitative analysis of the results. Additionally, none of the studies specifically focused on suicidality, which restricted the depth and clarity of the findings. The relatively small sample size of CHR-P participants with suicidality further limited the number of variables that could be analyzed within a single model, potentially affecting the generalizability of the results. The use of self-report questionnaires to assess suicidality also introduced the possibility of social desirability bias or exaggeration, especially among participants seeking help. Furthermore, the reliance on single retrospective assessments of suicidality may not fully capture the rapid and dynamic fluctuations in suicidal thoughts over time.

Moreover, although the authors included studies that employed gold-standard instruments for the assessment of Clinical High Risk for Psychosis, such as the SIPS and CAARMS, the studies selected for the evaluation of suicide risk did not utilize the Columbia Suicide Severity Rating Scale (C-SSRS), a tool currently considered the gold standard for assessing suicidality (47). The inclusion of studies employing this scale could have enriched the analysis and contributed to a more comprehensive understanding of suicide risk in CHR-P populations.

Finally, the prevalence and characteristics of suicidality within adolescent CHR-P populations have been insufficiently explored, highlighting the need for more focused and longitudinal studies to address these important gaps. Future studies should aim to refine diagnostic tools for better differentiating the various predictors of suicidality in CHR-P individuals, particularly in the adolescent population. Longitudinal research could help to clarify the trajectory of suicidality from the prodromal phase to full-blown psychosis and inform more effective, personalized interventions.
